# Screening and Evaluation of the Bioremediation Potential of Cu/Zn-Resistant, Autochthonous *Acinetobacter* sp. FQ-44 from *Sonchus oleraceus* L.

**DOI:** 10.3389/fpls.2016.01487

**Published:** 2016-09-30

**Authors:** Qing Fang, Zhengqiu Fan, Yujing Xie, Xiangrong Wang, Kun Li, Yafeng Liu

**Affiliations:** Department of Environmental Science and Engineering, Fudan UniversityShanghai, China

**Keywords:** *Sonchus oleraceus*, plant-growth-promoting rhizobacteria, Cu/Zn-resistant, bioremediation, *Acinetobacter*

## Abstract

The quest for new, promising and indigenous plant growth-promoting rhizobacteria and a deeper understanding of their relationship with plants are important considerations in the improvement of phytoremediation. This study focuses on the screening of plant beneficial Cu/Zn-resistant strains and assessment of their bioremediation potential (metal solubilization/tolerance/biosorption and effects on growth of *Brassica napus* seedlings) to identify suitable rhizobacteria and examine their roles in microbes-assisted phytoremediation. Sixty Cu/Zn-resistant rhizobacteria were initially isolated from *Sonchus oleraceus* grown at a multi-metal-polluted site in Shanghai, China. From these strains, 19 isolates that were all resistant to 300 mg⋅L^-1^ Cu as well as 300 mg⋅L^-1^ Zn, and could simultaneously grow on Dworkin–Foster salt minimal medium containing 1-aminocyclopropane-1-carboxylic acid were preliminarily selected. Of those 19 isolates, 10 isolates with superior plant growth-promoting properties (indole-3-acetic acid production, siderophore production, and insoluble phosphate solubilization) were secondly chosen and further evaluated to identify those with the highest bioremediation potential and capacity for bioaugmentation. Strain S44, identified as *Acinetobacter* sp. FQ-44 based on 16S rDNA sequencing, was specifically chosen as the most favorable strain owing to its strong capabilities to (1) promote the growth of rape seedlings (significantly increased root length, shoot length, and fresh weight by 92.60%, 31.00%, and 41.96%, respectively) under gnotobiotic conditions; (2) tolerate up to 1000 mg⋅L^-1^ Cu and 800 mg⋅L^-1^ Zn; (3) mobilize the highest concentrations of water-soluble Cu, Zn, Pb, and Fe (16.99, 0.98, 0.08, and 3.03 mg⋅L^-1^, respectively); and (4) adsorb the greatest quantities of Cu and Zn (7.53 and 6.61 mg⋅g^-1^ dry cell, respectively). Our findings suggest that *Acinetobacter* sp. FQ-44 could be exploited for bacteria-assisted phytoextraction. Moreover, the present study provides a comprehensive method for the screening of rhizobacteria for phytoremediation of multi-metal-polluted soils, especially those sewage sludge-amended soils contaminated with Cu/Zn.

## Introduction

Heavy metal pollution of soils has become a global environmental concern. Even essential biological trace elements, such as Zn and Cu, can be toxic or lethal to organisms at high concentrations (Ouzounidou, 1995). Unlike organic compounds, heavy metals in soils cannot be mineralized or broken down to less toxic forms (Chen et al., 2014). A large proportion of heavy metals are generally bound to organic and inorganic soil components or exist as insoluble precipitates, and are thus unavailable for root uptake by field-grown plants (Raskin et al., 1994). Therefore, developing appropriate strategies for the remediation of heavy-metal-polluted soils demands urgent attention from the perspectives of environmental conservation and human health (Aboushanab et al., 2006).

Phytoremediation, an emerging, challenging, and solar-driven *in situ* technology with lower cost and enhanced environmental friendliness in comparison to conventional physicochemical technologies, has received increasing attention from ecological researchers (Kumar et al., 1995). However, this plant-based technique is generally time-consuming, because most hyperaccumulators identified thus far are generally small-biomass and slow-growing (Rajkumar and Freitas, 2008a). Moreover, its efficiency is often limited by the metal bioavailability in soil, plant roots development, and plant tolerance to a particular metal (Pilon-Smits, 2005). Thus, developing alternative strategies that can improve the efficiency of phytoremediation are necessary.

Several researchers have suggested biotechnological approaches and proposed to incorporate plant-associated microorganisms (rhizospheric, endophytic bacteria, and mycorrhizal fungi) into phytoextraction systems (Rajkumar and Freitas, 2008a; Ma et al., 2009a; Sessitsch et al., 2013). In such systems, the plants and rhizosphere are two key factors that make phytoremediation a viable *in situ* technology. On one hand, the plants to be used for remediation of metal-polluted soils must be qualified with tolerance to at least one metal, high competitiveness, fast growth, and large biomass (Glick, 2010). On the other hand, the rhizosphere, as an important soil-plant interface, provides a complex dynamic microenvironment where root-associated microorganisms form unique communities that have a high potential to detoxify hazardous waste compounds (De Souza et al., 1999; Alford et al., 2010). Moreover, the particular microbial community with high activity and large contact area probably acts as a source of microbial chelates (Kärenlampi et al., 2000). Thus, the microorganism-assisted phytoremediation potential, as well as the mechanisms by which rhizobacteria enhance phytoremediation efficiency, has been attracting increasing research interest lately.

Among the plant-associated microbes, plant growth-promoting rhizobacteria (PGPR) are considered a major component of phytoremediation technology (Glick, 2003). They have capacity of plant growth-promoting (PGP) and improving phytoremediation by various mechanisms, including: fixation of atmospheric nitrogen, utilization of 1-aminocyclopropane-1-carboxylic acid (ACC), production of siderophores and antipathogenic substances, production of plant growth regulators, transformation of nutrient elements (Glick et al., 1999), bacteria-induced metal chelation (Adediran et al., 2015), and synthesis of cysteine-rich peptides (Adediran et al., 2016). Thus, inoculation with metal-resistant PGPR, particularly indigenous PGPR (Kozdrój et al., 2004), can improve the efficiency of heavy metal phytoremediation (Ma et al., 2011; Rajkumar et al., 2012). Therefore, researchers need to isolate and screen competitive and effective PGPR (Paau, 1989) that are well adapted to the conditions of a particular site (Sheng and Xia, 2006). Although PGPR play important roles in phytoremediation strategies, studies on Cu/Zn-resistant PGPR in this area remain very limited (Lucy et al., 2004), particularly field studies. Thus, more laboratory and field studies are needed to advance existing research.

*Sonchus oleraceus* is a cosmopolitan weed species native to Europe and central Asia (Hutchinson et al., 1984) that grows readily and adapts to diverse environments in many countries (Holm et al., 1977). In China, *S. oleraceus* is also widely distributed as an annual and roadside pioneer plant. It is one of few species found at disrupted locations, such as oil well sites in oilfields and barren lands (Xiong et al., 1997). Furthermore, *S. oleraceus* is regarded as the most suitable candidate for the removal of Zn and Cd from soils (Khan et al., 1998).

Despite numerous reports about rhizobacteria-enhanced phytoremediation of heavy metals (Sheng and Xia, 2006; Dell’Amico et al., 2008; Płociniczak et al., 2016), little information is available about effects Cu/Zn-resistant bacteria from the rhizosphere of *S. oleraceus* on plant growth and heavy metal bioavailability/biosorption in multi-metal-polluted soils. Thus, the quest for novel, beneficial and indigenous rhizobacteria among different plant species grown in multi-metal-polluted environments is very meaningful. In addition, to assess the potential rhizospheric mechanisms underlying the effects on plant growth and uptake and translocation of heavy metals, we explored the biochemical characteristics [production of indole-3-acetic acid (IAA), ACC deaminase (ACCD), and siderophores; and solubilization of inorganic phosphate] of selected bacteria. Furthermore, diverse genera of PGPR could affect plant growth in different ways, because the PGP effect could be plant- and/or PGPR-specific. Thus, our main objectives were to: (1) isolate and preliminarily screen Cu/Zn-resistant and ACCD-containing bacteria from the rhizosphere of *S. oleraceus* grown in multi-metal-polluted soils; (2) select indigenous PGPR with superior PGP traits that could effectively increase plant biomass under unfavorable conditions; and (3) evaluate the bioremediation potentials of different PGPR (Cu/Zn/Pb/Cd/Fe-solubilization, Cu/Zn-tolerance/biosorption and effects on the growth of rape) to identify more-suitable rhizobacteria and examine the effects of selected bacteria on plant growth and metal uptake/translocation in *Brassica napus* via sand culture experiments.

## Materials and Methods

### Sampling, Treatment, and Characterization of Soils and Plants

Soils were randomly sampled from a depth of 0–20 cm in the Jiading Wastewater Disposal Plant (31°22′32″ N, 121°09′57″ E), located at Shanghai, China. The soils used in this study were mixtures of sewage sludge and waste residue, and contaminated with multiple heavy metals. Before the experiments commenced, soil samples pretreatmented were air-dried for 1 month and sieved (4 mm) to remove as many plant materials, soil macrofauna, and stones as possible. The soil subsamples were then passed through a 2-mm stainless steel sieve, and subjected to physicochemical chatracterization according to standard methods (Lu, 1999), some of which are listed in **Table 1.**

**Table 1:** Physicochemical and microbiological properties of the tested soils.

Parameter	Data (means ± SE, *n* = 3)
Soil texture	Sandy loam soil
pH (H_2_O, 1:2 w/v)	7.71 ± 0.05
Cation exchange capacity (cmol⋅kg^-1^)	15.26 ± 0.03
Organic matter (%)	2.63 ± 0.02
Electric conductivity at 25°C (mS⋅cm^-1^)	3.04 ± 0.04
Total N (mg⋅kg^-1^)	1620.35 ± 113.00
Total Fe (mg⋅kg^-1^)	297.10 ± 0.13
Total Zn (mg⋅kg^-1^)	1263.78 ± 0.43
Total Pb (mg⋅kg^-1^)	153.26 ± 0.05
Total Ni (mg⋅kg^-1^)	65.21 ± 0.02
Total Cr (mg⋅kg^-1^)	206.61 ± 0.11
Total Cd (mg⋅kg^-1^)	3.03 ± 0.01
Total Hg (mg⋅kg^-1^)	0.31 ± 0.03
Total Cu (mg⋅kg^-1^)	650.10 ± 0.21
Total As (mg⋅kg^-1^)	11.54 ± 0.03
Total cultivable bacterial count^a^	2.55 ± 3.28 × 10^11^
Zn-resistant bacterial count	9.63 ± 2.08 × 10^8^
Cu-resistant bacterial count	7.79 ± 7.56 × 10^9^


*^a^Expressed as colony-forming units (CFU) per gram of fresh soil.*

Native *in situ S. oleraceus* plants were also randomly selected from the same wastewater disposal plant at which the multi-metal-polluted soils were collected. Soon after returning to the laboratory, the rhizospheric soils of *S. oleraceus* (2 cm radius around the roots) were collected by gently shaking the roots (Wenzel et al., 2003) to remove loosely attached soils and stored in a refrigerator at 4°C until further use.

### Isolation and Preliminary Screening of Cu/Zn-Resistant and ACC-Utilizing Rhizobacteria

Rhizobacteria were isolated from *S. oleraceus* according to the protocol of Jiang et al. (2008). Sixty pure isolates were initially isolated and stored in 30% (v/v) glycerol at –80°C until further analysis (Wei et al., 2009). Viable bacterial populations, including total and resistant bacteria were counted by the plate count method. The CFU/g of fresh soil is presented in **Table 1.** After isolation, all isolates were further streaked on two Luria–Bertani medium (LB) agar plates containing either Zn or Cu (100 to 500 mg⋅L^-1^, respectively) and monitored for growth. All plates were incubated in triplicate at 30°C for 48 h.

In order to obtain Cu/Zn-resistant PGPR, 46 isolates that were all simultaneously resistant to 300 mg⋅L^-1^ Cu and 300 mg⋅L^-1^ Zn were further tested for their ability to grow on Dworkin–Foster (DF) salt minimal medium containing ACC (denoted ADF) as a sole nitrogen source (Dworkin and Foster, 1958). The DF medium containing (NH_4_)_2_SO_4_ (Rajkumar and Freitas, 2008b) (denoted NDF) and without a nitrogen source were used as controls. We also analyzed the ACCD activity of cell-free extracts analyzed by quantifying the amount of α-ketobutyrate according to a modified method of Honma and Shimomura (1978). After preliminary screening, 19 isolates that were resistant to both 300 mg⋅L^-1^ Cu and 300 mg⋅L^-1^ Zn, and simultaneously growing on ADF were selected for further evaluation of PGP parameters (secondary screening).

### Evaluation of PGP Properties

Synthesis of IAA by the 19 isolates was quantified as described by Bric et al. (1991), using LB broth supplemented with 0.5 mg⋅mL^-1^ L-tryptophan. The IAA concentrations were calculated using a calibration curve of pure IAA as the standard (Sigma, USA). Bacterial siderophore production was detected and quantified by the chrome azurol S (CAS) analytical method (Schwyn and Neilands, 1987). According to this assay, the siderophore levels were defined as the A/A_r_ ratio and a smaller A/A_r_ ratio indicated higher siderophore output (Sheng et al., 2008). The phosphate-solubilizing ability of the isolates was analyzed in Pikovskaya’s medium (Pikovskaya, 1948) supplemented with 0.5% tricalcium phosphate. The soluble phosphate in the supernatant was quantified by the Mo-blue method (Watanabe and Olsen, 1965). After secondary screening, 10 functional strains with superior PGP traits were selected for further evaluation of bioremediation potential (the third screening).

### Evaluation of Bioremediation Potential by Functional Strains

#### Activation of Soil Metals by Functional Strains

Batch experiments on the effects of the 10 functional isolates on metal mobility in soil were conducted in triplicate 50-mL scaled polypropylene centrifuge tubes according to Chen et al. (2005). Briefly, pure cultures of functional strains were centrifuged at 8000 rpm for 10 min after 20 h of growth, washed twice in phosphate buffer (pH 7.0), and re-suspended in sterile distilled water. One milliliter of each washed bacterial suspension (OD_600_ = 1.0 ± 0.05) or sterile water (control) was added to the 1 g of autoclaved soils. All tubes were weighed, wrapped in brown paper, and placed on an orbital shaker at 180 rpm and 28°C. After 1 week, the tubes were weighed again to compensate for evaporation. Sterile water (10 mL) was then added to extract water-soluble metals. The soil suspensions were vibrated at 25°C for 2 h and centrifuged at 10,000 rpm for 10 min. The resulting supernatants were filtered through a 0.22-μm membrane filter for determination of pH and water-soluble Cu/Zn/Pb/Cd/Fe. The metal concentrations were determined by inductively coupled plasma-mass spectrometry (ICP-MS, SPECTRO).

#### Minimum Inhibitory Concentration (MIC) of Functional Strains

To check the extent of resistance, we used the secondly selected isolates to determine the lowest concentration of Cu and Zn that completely inhibited the growth of bacterial strains, termed as the minimum inhibitory concentration (MIC). Isolates were streaked in triplicate on LB agar media supplemented with varying concentrations (600 to 1000 mg⋅L^-1^) of Cu and Zn, respectively. For each strain and each metal, the lowest concentration that inhibited visible growth at 28°C within 3 days was determined.

### Metal Biosorption Analyses

The biosorption of Cu and Zn by bacterial cells was evaluated as described by Hernández et al. (1998) with some modifications. Bacterial cells obtained from the bacterial cultures (grown in LB broth at 28°C, OD_600_ = 1.0 ± 0.05) were harvested by centrifugation at 8000 rpm for 20 min and washed twice with sterile deionized water. The harvested cells were re-suspended in 150 mg⋅L^-1^ of Cu or Zn. An uninoculated solution was used as the control. After incubation at room temperature for 10 h, the cells were harvested following centrifugation and the residual metal ions in the supernatant were measured using a flame atomic absorption spectrophotometer (Varian Spectra model AA240FS; USA). The amount of metal absorbed by the bacterial cells was calculated by subtracting the metal concentration in the supernatant from the original concentration.

### *In vivo* Plant Growth Promotion Assay

Growth promotion of the secondly selected isolates was tested according to Patten and Glick (2002) with some modifications. Seeds of *B. napus* var. Zhongyou-1 were surface-sterilized with a mixture of absolute ethanol and 30% hydrogen peroxide (1:1, v/v) for 20 min, and washed twice with sterile distilled water before being transferred to sterile filter paper in a Petri dish. Seed sterility was monitored by incubating the seeds on LB agar at 30°C and aseptically placed on moistened filter paper. Then 6 mL of each bacterial suspension (OD_600_ = 0.5 ± 0.02) or sterile distilled water (uninoculated control) was added to glass Petri dishes with two-double filter paper. After incubation of closed Petri dishes for 7 days at 28°C in the dark, the root length, shoot length, fresh weight, and number of seedlings that had sprouted within 3 days were determined. The assay was performed twice with two dishes (10 seeds per dish) for each treatment. After the third screening, among the 10 functional strains, S44 with the highest bioremediation potentials was selected for genetic identification.

### Genetic Identification of S44

Genomic DNA of S44 was extracted as per a previously reported protocol (Araújo et al., 2002), and used as a template in 16S rDNA PCR amplification with universal primers 27F (5′-GAGTTTGATCACTGGCTCAG-3′) and 1492R (5′-TACGGCTACCTTGTTACGACTT-3′) (Byers et al., 1998). PCR amplification was performed in a DNA Engine Thermal Cycler (PTC-200, BioRad, USA) under the reaction conditions described by (Branco et al., 2005). The amplified product was purified with a DNA Purification Kit and sequenced at HuaDa Biotechnology Company (Shanghai, China). The partial 16S rDNA sequences obtained were matched with nucleotide sequences in GenBank using the BLAST tool^1^ Neighbor joining phylogenetic trees were constructed after calculation of a maximum composite likelihood distance matrix using the MEGA 4.0 software (Tamura et al., 2007).

### Sand Culture Experiment

Based on the results of the third screening, the *Acinetobacter* sp. FQ-44 was selected for preliminarily exploring roles of the plant-rhizobacteria partnership in heavy metal remediation. Surface-sterilized seeds of *B. napus* were pregerminated on sterile filter paper in a Petri dish. After germination (4 days), uniform seedlings were selected and soaked for 2 h in the bacterial culture (OD_600_ of 1.0 ± 0.05) or sterile water (control). Six seedlings were subsequently transplanted into a plastic pot (top diameter 85 mm, bottom diameter 65 mm, and height 105 mm) containing sterilized vermiculite and saturated with sterile half-strength Hoagland’s nutrient solution (Barac et al., 2004). One week after transplantation, seedlings were thinned to three per pot and subjected to various concentrations of Cu (2, 5, and 10 mg/L). Three replicates were conducted for each treatment. The plantlets were allowed to grow under greenhouse conditions (25 ± 5°C, 16:8 day/night regime). After 45 days, plants were carefully removed from the pots and root surfaces were immersed in 0.01 M EDTA for 30 min, and then rinsed thoroughly with deionized water to remove any surface adsorbed metals. Fresh and dry weights were measured and the concentrations of Cu in roots and shoots were determined using a flame atomic adsorption spectrophotometer. The translocation factor (TF) was calculated as the ratio of metal concentration in the shoots to that in the roots (Liu et al., 2009) and the bioaccumulation factor (BCF) was calculated as the ratio of metal contents in the entire plant to that in the soil (Bu-Olayan and Thomas, 2009).

### Statistical Analyses

Results for each treatment were expressed as means ± SD. Significant differences between parameters were tested using the *post hoc* Fisher’s protected least significant difference (LSD) test after one-way ANOVA. All statistical analyses, including the Pearson’s correlation analysis, were conducted using SPSS 18.0 (SPSS Inc., USA). Unless otherwise indicated, significant level was set at *P* < 0.05. Graphical analyses were performed on SigmaPlot 11.0 (Jandel Scientific, USA).

## Results and Discussion

### Isolation and Preliminary Screening of Cu/Zn-Tolerant and ACC-Utilizing Rhizobacteria

Before preliminary screening and identification, 60 cultivable isolates that were simultaneously resistant to 50 mg⋅L^-1^ of Zn and 50 mg⋅L^-1^ Cu, were isolated initially from the rhizosphere of *S. oleraceus* and named S1–S60. These bacterial isolates were autochthonous to the metal-polluted site and were thus more suitable for *in situ* phytoremediation of the multi-metal-polluted soils. As reported, rhizobacteria isolated from multi-metal-polluted natural environments can be constitutively or adaptively resistant to increasing metal concentrations, as they have adapted to such environments (Nies, 2003).

Soil microbes with generally higher metal resistance are the preferred choice for phytoremediation studies. Our results indicate that most of the isolates tested were resistant to different concentrations of Zn and Cu (**Supplementary Table S1**). Among all isolates, 34 were simultaneously resistant to 400 mg⋅L^-1^ Zn and 400 mg⋅L^-1^ Cu, among which some were even tolerant of 500 mg⋅L^-1^ Zn or Cu; whereas 46 isolates were able to simultaneously resist 300 mg⋅L^-1^ Zn and 300 mg⋅L^-1^ Cu. To obtain more plant beneficial strains, these 46 isolates were selected for further testing of their ACC utilization ability.

**FIGURE 1:**
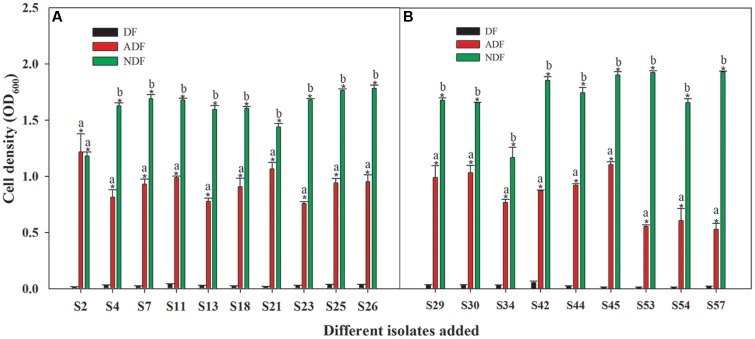
**Capacity for growth of 19 Cu/Zn-resistant isolates on ADF **(A,B)**.** Different small letters denote significant differences between treatments and an asterisk (^∗^) represents a significantly greater value on ADF and NDF compared to DF (*P* < 0.05).

Among those 46 isolates, 19 isolates grew significantly better on ADF and NDF than on DF (*P* < 0.05) (**Figure 1**). Although these isolates grew well on ADF and NDF, their growth without a nitrogen source was limited (**Figure 1**). Thus, these 19 rhizobacteria had the potential to utilize ACC as a sole nitrogen source. Moreover, they had the ability to grow on ADF to produce ACCD (**Figure 1**; **Supplementary Table S2**), which was supported by earlier observations that ACC-utilizing bacteria could generally produce ACCD. As reported, ACC-utilizing bacteria have been found to facilitate plant growth by producing ACCD that hydrolyzes the ethylene precursor ACC into α-ketobutyrate and ammonia (Glick, 2005) in the presence of salts or heavy metals (Belimov et al., 2005; Zahir et al., 2009). Consequently, these ACC-utilizing isolates could be important for PGPR-mediated phytoremediation.

### Screening of Functional Strains with Superior PGP Ability

Various PGP characteristics could contribute to reduced metal stress and increased growth in their host plants (Ma et al., 2011; Rajkumar et al., 2012). In our study, all 19 ACC-utilizing isolates had inherent abilities of IAA production, siderophore production, and insoluble phosphate solubilization (**Supplementary Table S2**). Out of 19 isolates, 10 with superior PGP traits were selected for statistical analyses (**Table 2**), because each had three indices that were all ranked in the top 10.

**Table 2:** PGP features of functional strains and pH of solutions in the phosphate solubilization experiment.

Functional strains	IAA synthesis (mg⋅L^-1^)	Siderophore production (A/A_r_)^a^	Phosphate solubilization (mg⋅L^-1^)^b^	pH
S21	10.55 ± 0.08d	0.13 ± 0.01ab	53.34 ± 1.29b	6.34 ± 0.18a
S23	6.48 ± 0.24bc	0.29 ± 0.01bcd	34.21 ± 1.06a	7.42 ± 0.03d
S25	7.01 ± 0.58c	0.40 ± 0.09d	36.36 ± 1.41a	6.97 ± 0.10bc
S26	3.45 ± 0.29a	0.25 ± 0.05abcd	35.00 ± 0.96a	7.17 ± 0.23cd
S29	4.42 ± 0.15ab	0.10 ± 0.01a	39.42 ± 1.38a	7.39 ± 0.03d
S30	5.10 ± 0.16abc	0.12 ± 0.05a	35.45 ± 1.06a	7.39 ± 0.07d
S42	20.17 ± 0.26e	0.34 ± 0.09cd	39.76 ± 0.82a	7.52 ± 0.01d
S44	29.57 ± 0.95g	0.29 ± 0.04bcd	74.75 ± 1.48c	6.76 ± 0.03b
S45	25.15 ± 0.56f	0.34 ± 0.02cd	55.38 ± 1.41b	7.19 ± 0.08cd
S57	9.81 ± 0.20d	0.23 ± 0.02abc	55.81 ± 1.55b	7.41 ± 0.01d


*Data of columns by the same letter are not significantly different between bacterial treatments according to the Fisher’s protected LSD test (*P* > 0.05).*

*^a^Siderophore production: little, 0.8–1.0; low, 0.6–0.8; moderate, 0.4–0.6; high, 0.2–0.4; very high, 0–0.2.*

*^b^Concentration of phosphorus.*

As shown in **Table 2**, S44, the best IAA producer (29.57 mg⋅L^-1^) in our study, produced significantly more IAA than the other nine strains (*P* < 0.05). As reported similarly, *Enterobacter ludwigii* BNM 0357 released about 30 μg IAA mL^-1^ (Shoebitz et al., 2009). In addition, the IAA production abilities of all 10 isolates might be within a reasonable range for observable PGP effects (Ma et al., 2009b) that might contribute to increased plant biomass. As reported, a low IAA production by PGP bacteria promotes primary root elongation, whereas a high level inhibit primary root growth (Xie et al., 1996). Our rape inoculation experiments also indicated the 10 moderate IAA producers were able to increase root length, which was generally promoted by IAA-producing rhizobacteria (Patten and Glick, 1996). Moreover, Pearson’s correlation analysis also revealed that IAA was significantly positively correlated with the fresh weight of seedlings (*r* = 0.70, *P* = 0.02).

Siderophores, another important PGPR-released metabolites, indirectly alleviate heavy metal toxicity by increasing the supply of iron to plants (Burd et al., 2000), thereby facilitating plant growth. In our study, siderophore production was highest in S29 among the 10 isolates, whereas it was lowest in S25 (**Table 2**). Furthermore, siderophores were responsible for the mobilization of insoluble metals such as Fe (**Table 3**) and were positively correlated with three growth parameters (**Supplementary Table S3**), although this was not significant (*P* > 0.05). Our results concurring with the earlier observations also show that siderophores produced by rhizosphere microorganisms could supply iron to plants via Fe-siderophore complexes under iron-limited conditions (Crowley et al., 1988) and inoculation with a siderophore-producing strain promotes plant growth (Tripathi et al., 2005).

**Table 3:** Correlations between solubilization factors and water-soluble heavy metals.

Correlations	Solubilization factors			Water-soluble heavy metals				
		
	pH	Phosphate solubilization	Siderphores	Cu	Zn	Pb	Cd	Fe
pH	1.00	-0.10	-0.42	0.47	-0.57	-0.06	-0.51	0.13
Phosphate solubilization	-0.10	1.00	0.07	0.44	0.50	0.47	0.26	0.54
Siderphores	-0.42	0.07	1.00	0.05	0.67	0.45	0.24	.08
Cu	0.47	0.44	0.05	1.00	0.39	0.06	-0.22	0.49
Zn	-0.57	0.50	0.67	0.39	1.00	0.31	0.34	0.37
Pb	-0.06	0.47	0.45	0.06	0.31	1.00	-0.24	0.00
Cd	-0.51	0.26	0.24	-0.22	0.34	-0.24	1.00	0.61
Fe	0.13	0.54	0.08	0.49	0.37	0.00	0.61	1.00


*Data of columns are pearson’s correlation coefficient.*

Another crucial PGP mechanism is phosphate solubilization, through which microbes enhance P availability to the host plant and thereby contribute to plant–bacteria interactions and PGP effects in metal-polluted soils (Zaidi et al., 2006). Our findings indicate that phosphate solubilization was positively correlated with all growth parameters (*r* = 0.45, 0.18, and 0.51 for root length, shoot length, and fresh weight, respectively) (**Supplementary Table S3**). Moreover, Rajkumar et al. (2009) also reported that phosphate solubilization in the rhizosphere greatly contributes to the PGP effects of bacteria. In addition, the highest phosphate-solubilizing ability was also observed in S44 (74.75 mg⋅L^-1^), which was significantly higher than other nine isolates (*P* < 0.05, **Table 2**).

The foregoing analyses indicate that these isolates were able to facilitate the growth of *B. napus* probably through these PGP traits. Consequently, the screening of soil bacteria with superior PGP abilities in a multi-metal-polluted environment is one key step in phytoremediation studies.

### Final Choice of S44

#### Effects of Functional Strains on the Mobility of Soil Metals

**FIGURE 2:**
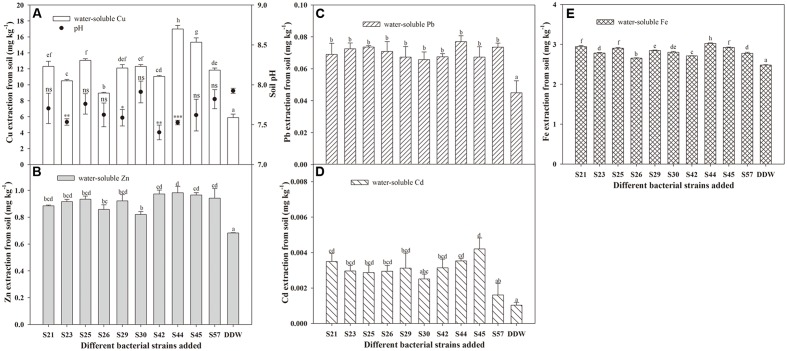
**Effects of inoculation with 10 isolates on mobilization of Cu **(A)**, Zn **(B)**, Pb **(C)**, Cd **(D)**, and Fe **(E)** in autoclaved soil.** Values are expressed as means ± SE, *n* = 9. ^∗^*P* < 0.05, ^∗∗^*P* < 0.01, ^∗∗∗^*P* < 0.001; ns, no significant difference. Different letters above the bar indicate significant differences among treatments at the level of *P* < 0.05 according to the Fisher’s protected LSD test.

Besides PGP traits, successful phytoremediation also depends mainly on metal bioavailability in the soil (Shallari et al., 2001). Therefore, to obtain effective metal-mobilizing strains, we further evaluated the ability of 10 isolates to increase water-soluble Cu, Zn, Cd, Pb, and Fe concentrations in soils. As expected, the presence of bacteria resulted in increased concentrations of water-extractable Cu, Zn, Pb, Cd, and Fe in autoclaved soil compared to axenic soil (**Figure 2**). These results suggest that the 10 Cu/Zn-resistant isolates had metal-solubilizing potential in heavy metal-polluted soil, thereby increasing metal bioavailability. As reported, Soil microorganisms can affect metal mobility and availability via the release of siderophores (Braud et al., 2009) and solubilization of metal phosphates (Aboushanab et al., 2006). Our results also indicate that siderophore and phosphate solubilization were both positively correlated with concentrations of water-soluble Cu, Zn, Pb, Cd, and Fe (**Table 3**).

Although all 10 isolates had the potential to facilitate the release of non-labile-phase Cu, Zn, Cd, Pb, and Fe from sterile soils, their effects actually differed (**Figure 2**). For example, the greatest amounts of water-soluble Cu, Zn, Pb, and Fe released in the soil were all found in S44, which were 16.99, 0.98, 0.08, 3.03 mg⋅L^-1^, respectively, but that of water-soluble Cd was observed in S45. Moreover, inoculation with S44 significantly increased the concentrations of water-soluble Cu, Zn, Pb, Cd, and Fe in soil by 1.88-, 0.44-, 0.71-, 2.50-, and 0.22-fold, respectively, compared to the control. Furthermore, the soil pH following inoculation with S44 dropped significantly compared to the control (*P* < 0.05; **Table 2**).

In addition, mobilization characteristics differed among the metals (**Figure 2**), which could be explained by the physicochemical properties of the various metals, metal-microbe interactions, as well as the unordered competition between metals. However, some isolates, such as S44 and S45, that exhibited high mobilization of one metal, were also remarkably capable of mobilizing other metals.

### MIC of Functional Strains

The preliminary resistance results showed that some isolates were able to grow in higher concentrations of all tested metals. Thus, to determine the extent of resistance, we assessed the Cu and Zn MICs of the secondly selected isolates. Our toxicity tests show that S26, S42, S44, S45, and S57 tolerated relatively high levels of Cu and Zn (**Table 4**). Moreover, among the 10 functional strains, S42, S44, and S45 had the highest Cu (1000 mg⋅L^-1^) and Zn (800 mg⋅L^-1^) MICs. This high tolerance of Cu and Zn could be attributed to the fact that these bacteria were isolated from the sewage-amended soils containing high levels of Cu and Zn. However, strain S21 was less tolerant of Cu (600 mg⋅L^-1^) and Zn (400 mg⋅L^-1^). In addition, the present results also indicate that Zn was more toxic to the isolates than Cu, which was different from some previous studies (Hassen et al., 1998; Jiang et al., 2008; Guo et al., 2011).

**Table 4:** MIC of the secondly selected rhizobacteria.

Metals	MIC (mg⋅L^-1^)									
	
	S21	S23	S25	S26	S29	S30	S42	S44	S45	S57
Zn	400	600	500	700	500	600	800	800	800	700
Cu	600	800	700	900	800	800	1000	1000	1000	800


### Metal Biosorption Potential of Secondly Selected Isolates

**FIGURE 3:**
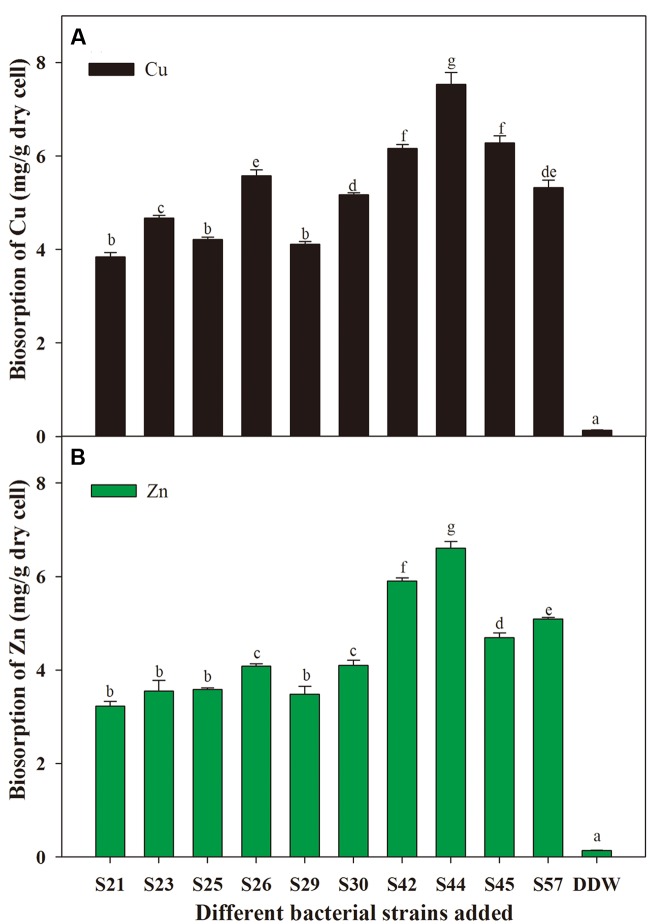
**Biosorption of Cu **(A)** and Zn **(B)** by secondly selected isolates.** Different letters above the bar indicate significant differences among treatments at the level of *P* < 0.05 according to the Fisher’s protected LSD test.

With respect to microbial remediation, it is very important to determine whether selected bacteria have the capacity for metal uptake. Our results indicate that different isolates exhibited different capacities for biosorption of the metal ions tested (**Figure 3**). Moreover, S44 exhibited the highest potentials to remove Cu (7.53 mg⋅g^-1^ dry cell) and Zn (6.61 mg⋅g^-1^ dry cell), and absorbed significantly more Cu and Zn than the other nine isolates (*P* < 0.05). Thus, application of the effective metal-solubilizing/absorbing S44 would be helpful for improving microbe-assisted phytoremediation. As reported, the biosorption capacity of bacteria plays an important role in reducing metal phytotoxicity by limiting the entry of metal ions into plant cells, and might contribute to enhanced plant growth in metal-contaminated soils (Ma et al., 2011). Furthermore, it should be noted that the biosorption ability for Cu was higher than that for Zn (**Figure 3**). One possible explanation could be that Cu (0.72 Å) with smaller ionic radius might be more rapidly complexed by bacterial cell wall/membrane compared to Zn (0.88 Å) (Karakagh et al., 2012). Another explanation probably was that Zn was more toxic to these isolates than Cu.

### Effects of Functional Strains on Rape Growth

After the 10 representative isolates infecting sterile *B. napus* L. seeds, seed germination was neither significantly inhibited nor stimulated. For example, seed germination after inoculation with S23 was equal to that of the control (**Table 5**).

**Table 5:** Growth parameters of *Brassica napus* L. seedlings in sterile filter paper following infection with 10 isolates.

Functionalstrains	Root length (cm)	Shoot length (cm)	Fresh weight (mg)^a^	Germination(%)	Vigor index^b^
Control	4.19 ± 0.58a	5.58 ± 0.12ab	73.40 ± 0.52a	80.00	7.85 ± 0.09a
S21	5.27 ± 0.66ab	6.44 ± 0.51abc	86.60 ± 1.39abc	82.50	9.63 ± 0.24b
S23	5.59 ± 0.55abc	7.52 ± 0.41c	94.90 ± 1.82bcd	80.00	10.49 ± 0.15bc
S25	5.37 ± 0.72ab	6.73 ± 0.79abc	82.10 ± 2.09ab	85.00	10.29 ± 0.20bc
S26	6.34 ± 0.71bcde	5.26 ± 0.28a	87.90 ± 0.96bc	90.00	10.44 ± 0.24bc
S29	6.37 ± 0.57bcde	7.56 ± 0.59c	97.10 ± 0.78bcd	87.50	12.19 ± 0.15de
S30	7.07 ± 0.34bcde	7.05 ± 0.83bc	93.13 ± 1.94bcd	87.50	12.35 ± 0.23ef
S42	7.37 ± 0.54cde	7.88 ± 0.55c	100.83 ± 1.79cd	77.50	11.82 ± 0.14cde
S44	8.07 ± 0.31e	7.31 ± 0.24c	104.20 ± 1.06d	90.00	13.84 ± 0.11fg
S45	5.84 ± 0.40abcd	6.68 ± 0.47abc	100.10 ± 1.30cd	85.00	10.63 ± 0.12 bcd
S57	7.68 ± 0.55de	7.96 ± 0.32c	95.60 ± 1.31bcd	90.00	14.08 ± 0.14h


*Values are expressed as means ± SE, *n* = 10. Different letters in the same column indicate significant differences among treatments at the level of *P* < 0.05 according to the Fisher’s protected LSD test.*

*^a^Fresh weight of seedling.*

*^b^Vigor index = germination (%) × seedling length (root length + shoot length).*

A deeper understanding of plant-microbe interactions is complicated, but applicable to microbe-assisted phytoreme diation. In our study, seeds inoculated with the various isolates all had longer roots compared to the control (**Table 5**). Moreover, the most significant increase in root length was observed with S44 (92.60%, *P* < 0.05). Although the maximum shoot elongation was observed with S57, inoculation with S44 significantly increased shoot length by 31.00% (*P* < 0.05), compared to the control. Furthermore, the maximum promoting effect on fresh weight was also observed with S44, showing a significant increase by 41.96% (*P* < 0.05; **Table 5**). In addition, the highest seed vigor index was observed with S57 followed by S44 and S30, all exhibiting significant effects (*P* < 0.05). The foregoing results indicate that S44 has higher potential to facilitate the growth of *B. napus*.

Although the selected isolates showed PGP effects, these responses were not evaluated in the presence of metal stress, which would more effectively demonstrate PGPR-mediated phytoremediation. Of the 10 functional strains, S44 was selected as the most active strain (tolerance of up to 800 mg⋅L^-1^ Zn and 1000 mg⋅L^-1^ Cu, adsorption/solubilization of the largest quantities of Cu and Zn, the maximum root length and fresh weight-promoting effects) for molecular identification.

### Molecular Identification of Strain S44

**FIGURE 4:**
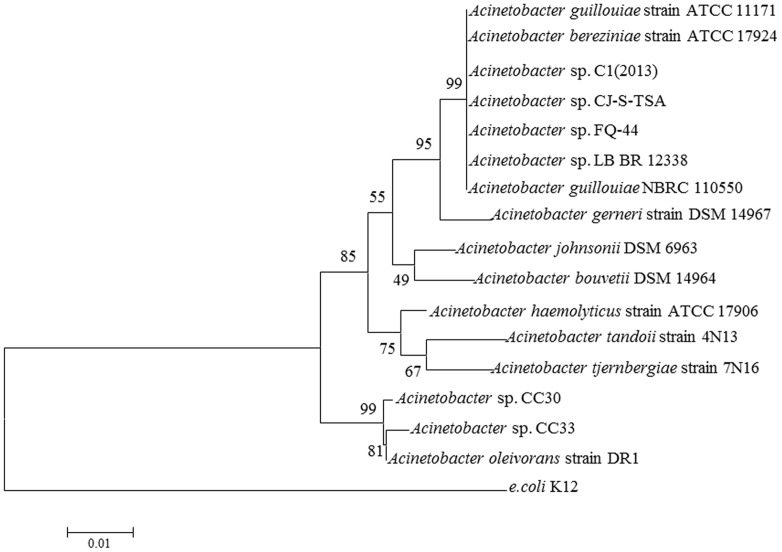
**Neighbor joining phylogenetic tree analysis of *Acinetobacter* sp.** FQ-44 with closely related strains from GenBank and relevant reports. The scale bar represents 0.01 substitutions per site.

S44 was identified as a species of *Acinetobacter* sp. by 16S rDNA gene sequencing and was named *Acinetobacter* sp. FQ-44. The highest sequence similarity (99%) and the phylogenetic tree in **Figure 4**, based on 16S rDNA sequences reveal a relationship between FQ-44 and other relevant bacteria reported. The 16S rDNA sequences (1443 bp) of FQ-44 were deposited in GenBank under accession No. KU206487.

### Influence of FQ-44 on Growth and Cu Uptake by *B. napus*

**FIGURE 5:**
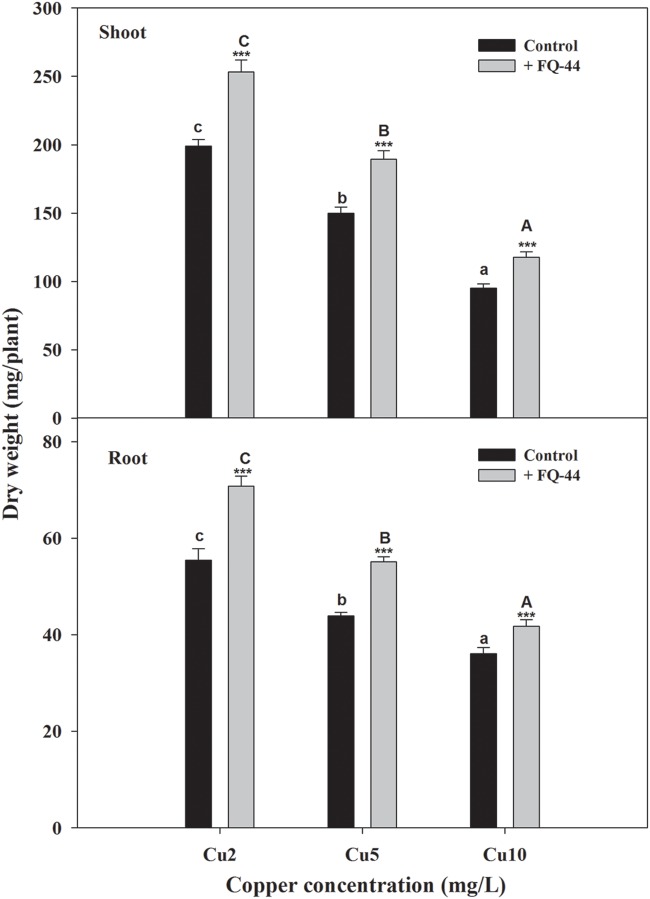
**Effects of FQ-44 on dry weight of root and shoot in *Brassica napus* cultivated in different concentrations of Cu.** Values are expressed as means ± SE, *n* = 9, ^∗∗∗^*P* < 0.001. Different letters above the bar indicate significant differences within the same microbial treatments (control and inoculation) at the level of *P* < 0.05, according to the Fisher’s protected LSD test. Cu2, 2 mg/L Cu; Cu5, 5 mg/L Cu; Cu10, 10 mg/L Cu.

The plant–bacteria partnership can be applied to increase the phytoremediation efficiency of soil and water contaminated with organic and/or inorganic pollutants (Khan et al., 2015). Therefore, the effects of metal-mobilizing FQ-44 on growth and metal uptake/translocation by *B. napus* were evaluated. As expected, FQ-44 significantly increased the dry weight of *B. napus* cultivated in different concentrations of Cu (**Figure 5**). In general, inoculation with FQ-44 significantly increased plant uptake of Cu (**Table 6**), which is consistent with significant improvements of BCF of Cu induced by FQ-44. Moreover, FQ-44 also significantly increased the TF of Cu (*P* < 0.05, **Table 6**), besides the Cu concentration of 2 mg/L. Yoon et al. (2006) also demonstrated that plants with a greater BCF and TF have the potential for use in heavy metal phytoextraction. The above results suggest that FQ-44 can be used to facilitate the phytoextraction of Cu. Previously, Rojas-Tapias et al. (2012) also reported that *Acinetobacter* sp. CC30 significantly enhanced Cu uptake by sunflowers. Moreover, Jing et al. (2014) reported that *Enterobacter* sp. JYX7 and *Klebsiella* sp. JYX10 significantly improved Zn uptake by *B. napus*. Recently, Płociniczak et al. (2016) also reported that *Brevibacterium casei* MH8a colonized white mustard plant tissues and enhanced Cu and Zn phytoextraction.

**Table 6:** Effects of FQ-44 on accumulation, uptake, BCF, and TF of Cu in *B. napus* cultivated in the presence of Cu at various concentrations.

Microbialtreatments	Cutreatments (mg/L)	Cu rootconcentrations (mg/kg DW)	Cu shootconcentrations (mg/kg DW)	TF	Cu root contents (μg)	Cu shoot contents (μg)	BCF
Control	2	74.39 ± 2.61a	51.36 ± 1.34a	0.69	4.15 ± 0.18a	10.14 ± 0.13a	0.48
	5	165.14 ± 3.62b	81.32 ± 2.07b	0.49	7.30 ± 0.13b	11.91 ± 0.19b	0.38
	10	258.67 ± 4.34c	126.47 ± 3.26c	0.49	9.34 ± 0.33c	12.06 ± 0.30b	0.21
FQ-44	2	77.22 ± 2.93nsA	53.44 ± 1.26nsA	0.69ns	5.47 ± 0.16***A	13.53 ± 0.14***A	0.63^∗∗^
	5	161.58 ± 3.75nsB	85.78 ± 3.69*B	0.53^∗^	8.89 ± 0.18***B	16.47 ± 0.26***B	0.50^∗∗^
	10	239.11 ± 4.80**C	155.41 ± 3.25***C	0.65^∗∗∗^	9.94 ± 0.22nsC	18.26 ± 0.38***C	0.28^∗^


*Values are the means ± SE, *n* = 9. The asterisk (^∗^) denotes a significant difference compared to the control treatment. ^ns^*p* > 0.05, ^∗^*p* < 0.05, ^∗∗^*p* < 0.01, ^∗∗∗^*p* < 0.001. Data of columns indexed by the different letters within the same microbial treatments (control and inoculation) are significantly different according to Fisher’s protected LSD test (*p* < 0.05).*

Although FQ-44 showed PGP effects on rape and enhanced phytoextraction of Cu, its colonization and survival properties are crucial features to evaluate its capacity for promoting sustainable plant growth and cope with metal stress in contaminated sites (Ma et al., 2011). Therefore, future studies using pot experiments containing *in situ* soils are needed to examine the specific effects of selected FQ-44 on the growth of host plants, and to determine whether it has the advantage of rhizosphere colonization.

## Conclusion

In the present study, the selection of Cu/Zn-resistant FQ-44 isolated from *S. oleraceus* was evaluated through three inter-causal screenings. Our results indicate that FQ-44 has potential to facilitate *B. napus* growth and enhance phytoextraction of Cu by sand culture experiment, which could be attributed to beneficial PGP traits; increased concentrations of water-soluble Cu, Cd, Zn, Pb, and Fe; and tolerance and adsorption of Cu and Zn that effectively improved microbe-assisted phytoremediation. Consequently, these advantages confer bioinoculant properties to FQ-44 that would be helpful for enhancing phytoremediation efficiency of multi-metal-polluted soils, particularly Cu/Zn-contaminated soils. Moreover, the proposed approach to screening in the present study could be useful for the isolation of effective strains and improvement of phytoremediation.

Although FQ-44 possessed PGP traits to facilitate *B. napus* growth and critical bioremediation potentials, in many cases PGP bacteria failed to induce the desired effects, when applied in a natural environment. Further research will address: (1) the interactions between FQ-44 and host plants; (2) the colonization potential of FQ-44 and mechanisms contributing to increased plant biomass and metal uptake/translocation by pot experiment containing *in situ* soils; and (3) the roles of FQ-44 in field phytoremediation experiments.

## Author Contributions

Conceived and designed the experiments: XW, QF, ZF, and YX. Conducted the work: QF. Analyzed the data: XW, QF, ZF, and YX. Contributed reagents/materials/analysis tools: XW, ZF, YX, and KL, YL. Wrote the manuscript: QF.

## Conflict of Interest Statement

The authors declare that the research was conducted in the absence of any commercial or financial relationships that could be construed as a potential conflict of interest.

## Footnotes

1 www.ncbi.nlm.nih.gov.

## References

[B1] Aboushanab R. Angle J. Chaney R. (2006). Bacterial inoculants affecting nickel uptake by *Alyssum murale* from low, moderate and high Ni soils. *Soil Biol. Biochem.* 38 2882–2889. 10.1016/j.soilbio.2006.04.045

[B2] Adediran G. A. Ngwenya B. T. Mosselmans J. F. W. Heal K. V. (2016). Bacteria-zinc co-localization implicates enhanced synthesis of cysteine-rich peptides in zinc detoxification when *Brassica juncea* is inoculated with *Rhizobium leguminosarum*. *New Phytol.* 209 280–293. 10.1111/nph.13588 26263508 PMC4676334

[B3] Adediran G. A. Ngwenya B. T. Mosselmans J. F. W. Heal K. V. Harvie B. A. (2015). Mechanisms behind bacteria induced plant growth promotion and Zn accumulation in *Brassica juncea*. *J. Hazard. Mater.* 283 490–499. 10.1016/j.jhazmat.2014.09.064 25464287

[B4] Alford É. R. Pilon-Smits E. A. Paschke M. W. (2010). Metallophytes-a view from the rhizosphere. *Plant Soil* 337 33–50. 10.1007/s11104-010-0482-3

[B5] Araújo W. L. Marcon J. Maccheroni W. van Elsas J. D. van Vuurde J. W. Azevedo J. L. (2002). Diversity of endophytic bacterial populations and their interaction with *Xylella fastidiosa* in citrus plants. *Appl. Environ. Microbiol.* 68 4906–4914. 10.1128/AEM.68.10.4906-4914.2002 12324338 PMC126398

[B6] Barac T. Taghavi S. Borremans B. Provoost A. Oeyen L. Colpaert J. V. (2004). Engineered endophytic bacteria improve phytoremediation of water-soluble, volatile, organic pollutants. *Nat. Biotechnol.* 22 583–588. 10.1038/nbt960 15077119

[B7] Belimov A. Hontzeas N. Safronova V. Demchinskaya S. Piluzza G. Bullitta S. (2005). Cadmium-tolerant plant growth-promoting bacteria associated with the roots of Indian mustard (*Brassica juncea* L. Czern.). *Soil Biol. Biochem.* 37 241–250. 10.1016/j.soilbio.2004.07.033

[B8] Branco R. Chung A. P. Veríssimo A. Morais P. V. (2005). Impact of chromium-contaminated wastewaters on the microbial community of a river. *FEMS Microbiol. Ecol.* 54 35–46. 10.1016/j.femsec.2005.02.014 16329970

[B9] Braud A. Jézéquel K. Bazot S. Lebeau T. (2009). Enhanced phytoextraction of an agricultural Cr- and Pb-contaminated soil by bioaugmentation with siderophore-producing bacteria. *Chemosphere* 74 280–286. 10.1016/j.chemosphere.2008.09.013 18945474

[B10] Bric J. M. Bostock R. M. Silverstone S. E. (1991). Rapid in situ assay for indole acetic acid production by bacteria immobilized on a nitrocellulose membrane. *Appl. Environ. Microbiol.* 57 535–538. 16348419 10.1128/aem.57.2.535-538.1991 PMC182744

[B11] Bu-Olayan A. H. Thomas B. V. (2009). Translocation and bioaccumulation of trace metals in desert plants of Kuwait Governorates. *Res. J. Environ. Sci.* 3 581–587. 10.3923/rjes.2009.581.587

[B12] Burd G. I. Dixon D. G. Glick B. R. (2000). Plant growth-promoting bacteria that decrease heavy metal toxicity in plants. *Can. J. Microbiol.* 46 237–245. 10.1139/w99-143 10749537

[B13] Byers H. K. Stackebrandt E. Hayward C. Blackall L. L. (1998). Molecular investigation of a microbial mat associated with the great artesian basin. *FEMS Microbiol. Ecol.* 25 391–403. 10.1111/j.1574-6941.1998.tb00491.x

[B14] Chen L. Luo S. L. Li X. J. Wan Y. Chen J. L. Liu C. B. (2014). Interaction of Cd-hyperaccumulator *Solanum nigrum* L. and functional endophyte *Pseudomonas* sp. Lk9 on soil heavy metals uptake. *Soil Biol. Biochem.* 68 300–308. 10.1016/j.soilbio.2013.10.021

[B15] Chen Y. X. Wang Y. P. Lin Q. Luo Y. M. (2005). Effect of copper-tolerant rhizosphere bacteria on mobility of copper in soil and copper accumulation by *Elsholtzia splendens*. *Environ. Int.* 31 861–866. 10.1016/j.envint.2005.05.044 16005516

[B16] Crowley D. E. Reid C. P. Szaniszlo P. J. (1988). Utilization of microbial siderophores in iron acquisition by oat. *Plant Physiol.* 87 680–685. 10.1104/pp.87.3.680 16666207 PMC1054820

[B17] De Souza M. Huang C. Chee N. Terry N. (1999). Rhizosphere bacteria enhance the accumulation of selenium and mercury in wetland plants. *Planta* 209 259–263. 10.1007/s004250050630 10436229

[B18] Dell’Amico E. Cavalca L. Andreoni V. (2008). Improvement of *Brassica napus* growth under cadmium stress by cadmium-resistant rhizobacteria. *Soil Biol. Biochem.* 40 74–84. 10.1016/j.soilbio.2007.06.024

[B19] Dworkin M. Foster J. (1958). Experiments with some microorganisms which utilize ethane and hydrogen. *J. Bacteriol.* 75 592–603. 13538930 10.1128/jb.75.5.592-603.1958 PMC290115

[B20] Glick B. R. (2003). Phytoremediation: synergistic use of plants and bacteria to clean up the environment. *Biotechnol. Adv.* 21 383–393. 10.1016/S0734-9750(03)00055-7 14499121

[B21] Glick B. R. (2005). Modulation of plant ethylene levels by the bacterial enzyme ACC deaminase. *FEMS Microbiol. Lett.* 251 1–7. 10.1016/j.femsle.2005.07.030 16099604

[B22] Glick B. R. (2010). Using soil bacteria to facilitate phytoremediation. *Biotechnol. Adv.* 28 367–374. 10.1016/j.biotechadv.2010.02.001 20149857

[B23] Glick B. R. Patten C. L. Holguin G. Penrose D. (1999). *Biochemical and Genetic Mechanisms Used by Plant Growth Promoting Bacteria.* London: Imperial College Press.

[B24] Guo J. Tang S. Ju X. Ding Y. Liao S. Song N. (2011). Effects of inoculation of a plant growth promoting rhizobacterium *Burkholderia* sp. *D*54 on plant growth and metal uptake by a hyperaccumulator *Sedum alfredii* Hance grown on multiple metal contaminated soil. *World J. Microbiol. Biotechnol.* 27 2835–2844. 10.1007/s11274-011-0762-y

[B25] Hassen A. Saidi N. Cherif M. Boudabous A. (1998). Resistance of environmental bacteria to heavy metals. *Bioresour. Technol.* 64 7–15. 10.1016/S0960-8524(97)00161-2

[B26] Hernández A. Mellado R. P. Martínez J. L. (1998). Metal accumulation and vanadium-induced multidrug resistance by environmental isolates of *Escherichia hermannii* and *Enterobacter cloacae*. *Appl. Environ. Microbiol.* 64 4317–4320. 9797283 10.1128/aem.64.11.4317-4320.1998 PMC106645

[B27] Holm L. Plunknett D. Poncho J. Herberger J. (1977). *The World’s Worst Weeds: Distribution and Biology.* Honolulu, HI: The University Press of Hawaii.

[B28] Honma M. Shimomura T. (1978). Metabolism of 1-aminocyclopropane-1-carboxylic acid. *Agric. Biol. Chem.* 42 1825–1831. 10.1271/bbb1961.42.1825

[B29] Hutchinson I. Colosi J. Lewin R. A. (1984). The biology of canadian weeds, 63: *Sonchus asper* (L.) Hill and *S. oleraceus* L. *Can. J. Plant Sci.* 64 731–744. 10.4141/cjps84-100

[B30] Jiang C. Y. Sheng X. F. Qian M. Wang Q. Y. (2008). Isolation and characterization of a heavy metal-resistant *Burkholderia* sp. from heavy metal-contaminated paddy field soil and its potential in promoting plant growth and heavy metal accumulation in metal-polluted soil. *Chemosphere* 72 157–164. 10.1016/j.chemosphere.2008.02.006 18348897

[B31] Jing Y. X. Yan J. L. He H. D. Yang D. J. Xiao L. Zhong T. (2014). Characterization of bacteria in the rhizosphere soils of *Polygonum* pubescens and their potential in promoting growth and Cd, Pb, Zn uptake by *Brassica napus*. *Int. J. Phytoremediation* 16 321–333. 10.1080/15226514.2013.773283 24912234

[B32] Karakagh R. M. Chorom M. Motamedi H. Kalkhajeh Y. K. Oustan S. (2012). Biosorption of Cd and Ni by inactivated bacteria isolated from agricultural soil treated with sewage sludge. *Ecohydrol. Hydrobiol.* 12 191–198. 10.1016/S1642-3593(12)70203-3

[B33] Kärenlampi S. Schat H. Vangronsveld J. Verkleij J. van der Lelie D. Mergeay M. (2000). Genetic engineering in the improvement of plants for phytoremediation of metal polluted soils. *Environ. Pollut.* 107 225–231. 10.1016/S0269-7491(99)00141-4 15092999

[B34] Khan A. Chaudhry T. Hayes W. Khoo C. Hill L. Fernandez R. (1998). Physical, chemical and biological characterisation of a steelworks waste site at Port Kembla, NSW, Australia. *Water Air Soil Pollut.* 104 389–402. 10.1023/A:1004951530917

[B35] Khan M. U. Sessitsch A. Harris M. Fatima K. Imran A. Arslan M. (2015). Cr-resistant rhizo- and endophytic bacteria associated with *Prosopis juliflora* and their potential as phytoremediation enhancing agents in metal-degraded soils. *Front. Plant Sci.* 5:755 10.3389/fpls.2014.00755 PMC4284999 25610444

[B36] Kozdrój J. Trevors J. Van Elsas J. (2004). Influence of introduced potential biocontrol agents on maize seedling growth and bacterial community structure in the rhizosphere. *Soil Biol. Biochem.* 36 1775–1784. 10.1016/j.soilbio.2004.04.034

[B37] Kumar P. N. Dushenkov V. Motto H. Raskin I. (1995). Phytoextraction: the use of plants to remove heavy metals from soils. *Environ. Sci. Technol.* 29 1232–1238. 10.1021/es00005a014 22192016

[B38] Liu Z. He X. Chen W. Yuan F. Yan K. Tao D. (2009). Accumulation and tolerance characteristics of cadmium in a potential hyperaccumulator-*Lonicera japonica* Thunb. *J. Hazard. Mater.* 169 170–175. 10.1016/j.jhazmat.2009.03.090 19380199

[B39] Lu R. (1999). *Analytical Methods for Soils and Agricultural Chemistry.* Beijing: China Agricultural Science and Technology Press.

[B40] Lucy M. Reed E. Glick B. R. (2004). Applications of free living plant growth-promoting rhizobacteria. *Antonie Van Leeuwenhoek* 86 1–25. 10.1023/B:ANTO.0000024903.10757.6e 15103234

[B41] Ma Y. Prasad M. Rajkumar M. Freitas H. (2011). Plant growth promoting rhizobacteria and endophytes accelerate phytoremediation of metalliferous soils. *Biotechnol. Adv.* 29 248–258. 10.1016/j.biotechadv.2010.12.001 21147211

[B42] Ma Y. Rajkumar M. Freitas H. (2009a). Improvement of plant growth and nickel uptake by nickel resistant-plant-growth promoting bacteria. *J. Hazard. Mater.* 166 1154–1161. 10.1016/j.jhazmat.2008.12.018 19147283

[B43] Ma Y. Rajkumar M. Freitas H. (2009b). Isolation and characterization of Ni mobilizing PGPB from serpentine soils and their potential in promoting plant growth and Ni accumulation by *Brassica* spp. *Chemosphere* 75 719–725. 10.1016/j.chemosphere.2009.01.056 19232424

[B44] Nies D. H. (2003). Efflux-mediated heavy metal resistance in prokaryotes. *FEMS Microbiol. Rev.* 27 313–339. 10.1016/S0168-6445(03)00048-2 12829273

[B45] Ouzounidou G. (1995). Cu-ions mediated changes in growth, chlorophyll and other ion contents in a Cu-tolerant *Koeleria splendens*. *Biol. Plant.* 37 71–78. 10.1007/BF02913000

[B46] Paau A. S. (1989). Improvement of *Rhizobium* inoculants. *Appl. Environ. Microbiol.* 55 862–865. 16347891 10.1128/aem.55.4.862-865.1989 PMC184215

[B47] Patten C. L. Glick B. R. (1996). Bacterial biosynthesis of indole-3-acetic acid. *Can. J. Microbiol.* 42 207–220. 10.1139/m96-032 8868227

[B48] Patten C. L. Glick B. R. (2002). Role of *Pseudomonas* putida indole acetic acid in development of the host plant root system. *Appl. Environ. Microbiol.* 68 3795–3801. 10.1128/AEM.68.8.3795-3801.2002 12147474 PMC124051

[B49] Pikovskaya R. (1948). Mobilization of phosphorus in soil in connection with vital activity of some microbial species. *Mikrobiologiya* 17 362–370.

[B50] Pilon-Smits E. (2005). Phytoremediation. *Ann. Rev. Plant Biol.* 56 15–39. 10.1146/annurev.arplant.56.032604.144214 15862088

[B51] Płociniczak T. Sinkkonen A. Romantschuk M. Sułowicz S. Piotrowska-Seget Z. (2016). Rhizospheric bacterial strain *Brevibacterium casei* MH8a colonizes plant tissues and enhances Cd, Zn, Cu phytoextraction by white mustard. *Front. Plant Sci.* 7:101 10.3389/fpls.2016.00101 PMC4754770 26909087

[B52] Rajkumar M. Ae N. Freitas H. (2009). Endophytic bacteria and their potential to enhance heavy metal phytoextraction. *Chemosphere* 77 153–160. 10.1016/j.chemosphere.2009.06.047 19647283

[B53] Rajkumar M. Freitas H. (2008a). Effects of inoculation of plant-growth promoting bacteria on Ni uptake by Indian mustard. *Bioresour. Technol.* 99 3491–3498. 10.1016/j.biortech.2007.07.046 17826991

[B54] Rajkumar M. Freitas H. (2008b). Influence of metal resistant-plant growth-promoting bacteria on the growth of *Ricinus communis* in soil contaminated with heavy metals. *Chemosphere* 71 834–842. 10.1016/j.chemosphere.2007.11.038 18164365

[B55] Rajkumar M. Sandhya S. Prasad M. Freitas H. (2012). Perspectives of plant-associated microbes in heavy metal phytoremediation. *Biotechnol. Adv.* 30 1562–1574. 10.1016/j.biotechadv.2012.04.011 22580219

[B56] Raskin I. Kumar P. N. Dushenkov S. Salt D. E. (1994). Bioconcentration of heavy metals by plants. *Curr. Opin. Biotechnol.* 5 285–290. 10.1016/0958-1669(94)90030-2

[B57] Rojas-Tapias D. F. Bonilla R. R. Dussán J. (2012). Effect of inoculation with plant growth-promoting bacteria on growth and copper uptake by sunflowers. *Water Air Soil Pollut.* 223 643–654. 10.1016/j.jhazmat.2011.02.075

[B58] Schwyn B. Neilands J. (1987). Universal chemical assay for the detection and determination of siderophores. *Anal. Biochem.* 160 47–56. 10.1016/0003-2697(87)90612-9 2952030

[B59] Sessitsch A. Kuffner M. Kidd P. Vangronsveld J. Wenzel W. W. Fallmann K. (2013). The role of plant-associated bacteria in the mobilization and phytoextraction of trace elements in contaminated soils. *Soil Biol. Biochem.* 60 182–194. 10.1016/j.soilbio.2013.01.012 23645938 PMC3618436

[B60] Shallari S. Echevarria G. Schwartz C. Morel J. (2001). Availability of nickel in soils for the hyperaccumulator *Alyssum murale* (Waldst & Kit). *South Afr. J. Sci.* 97(11/12; PART 2), 568–570.

[B61] Sheng X. F. Xia J. J. (2006). Improvement of rape (*Brassica napus*) plant growth and cadmium uptake by cadmium-resistant bacteria. *Chemosphere* 64 1036–1042. 10.1016/j.chemosphere.2006.01.051 16516946

[B62] Sheng X. F. Xia J. J. Jiang C. Y. He L. Y. Qian M. (2008). Characterization of heavy metal-resistant endophytic bacteria from rape (*Brassica napus*) roots and their potential in promoting the growth and lead accumulation of rape. *Environ. Pollut.* 156 1164–1170. 10.1016/j.envpol.2008.04.007 18490091

[B63] Shoebitz M. Ribaudo C. M. Pardo M. A. Cantore M. L. Ciampi L. Curá J. A. (2009). Plant growth promoting properties of a strain of *Enterobacter ludwigii* isolated from *Lolium perenne* rhizosphere. *Soil Biol. Biochem.* 41 1768–1774. 10.1016/j.soilbio.2007.12.031

[B64] Tamura K. Dudley J. Nei M. Kumar S. (2007). MEGA4: molecular evolutionary genetics analysis (MEGA) software version 4.0. *Mol. Biol. Evol.* 24 1596–1599. 10.1093/molbev/msm092 17488738

[B65] Tripathi M. Munot H. P. Shouche Y. Meyer J. M. Goel R. (2005). Isolation and functional characterization of siderophore-producing lead-and cadmium-resistant *Pseudomonas putida* KNP9. *Curr. Microbiol.* 50 233–237. 10.1007/s00284-004-4459-4 15886913

[B66] Watanabe F. Olsen S. (1965). Test of an ascorbic acid method for determining phosphorus in water and NaHCO3 extracts from soil. *Soil Sci. Soc. Am. J.* 29 677–678. 10.2136/sssaj1965.03615995002900060025x

[B67] Wei G. Chen W. Zhu W. Chen C. Young J. P. W. Bontemps C. (2009). Invasive *Robinia pseudoacacia* in China is nodulated by *Mesorhizobium* and *Sinorhizobium* species that share similar nodulation genes with native American symbionts. *FEMS Microbiol. Ecol.* 68 320–328. 10.1111/j.1574-6941.2009.00673.x 19416352

[B68] Wenzel W. Bunkowski M. Puschenreiter M. Horak O. (2003). Rhizosphere characteristics of indigenously growing nickel hyperaccumulator and excluder plants on serpentine soil. *Environ. Pollut.* 123 131–138. 10.1016/S0269-7491(02)00341-X 12663213

[B69] Xie H. Pasternak J. Glick B. R. (1996). Isolation and characterization of mutants of the plant growth-promoting rhizobacterium *Pseudomonas putida* GR12-2 that overproduce indoleacetic acid. *Curr. Microbiol.* 32 67–71. 10.1007/s002849900012

[B70] Xiong Z. T. Hu H. X. Wang Y. X. Fu G. H. Tan Z. Q. Yan G. A. (1997). Comparative analyses of soil contaminant levels and plant species diversity at developing and disused oil well sites in Qianjiang oilfield, China. *Bull. Environ. Contam. Toxicol.* 58 667–672. 10.1007/s001289900385 9060387

[B71] Yoon J. Cao X. Zhou Q. Ma L. Q. (2006). Accumulation of Pb, Cu, and Zn in native plants growing on a contaminated Florida site. *Sci. Total Environ.* 368 456–464. 10.1016/j.scitotenv.2006.01.016 16600337

[B72] Zahir Z. A. Ghani U. Naveed M. Nadeem S. M. Asghar H. N. (2009). Comparative effectiveness of *Pseudomonas* and *Serratia* sp. containing ACC-deaminase for improving growth and yield of wheat (*Triticum aestivum* L.) under salt-stressed conditions. *Arch. Microbiol.* 191 415–424. 10.1007/s00203-009-0466-y 19255743

[B73] Zaidi S. Usmani S. Singh B. R. Musarrat J. (2006). Significance of *Bacillus subtilis* strain SJ-101 as a bioinoculant for concurrent plant growth promotion and nickel accumulation in *Brassica juncea*. *Chemosphere* 64 991–997. 10.1016/j.chemosphere.2005.12.057 16487570

