# Supplementary material for: Screening and Evaluation of the Bioremediation Potential of Cu/Zn-Resistant, Autochthonous Acinetobacter sp. FQ-44 from Sonchus oleraceus L
Source: Front Plant Sci. 2016 Sep 30;7:1487. doi: 10.3389/fpls.2016.01487 (PMC5043060; doi:10.3389/fpls.2016.01487)
Supplement: Supplementary file 1 [file Table_1.DOCX]

**Table S1.** **Evaluation of heavy metal resistance for all isolates.**

| Isolates | Zn (mg/L) | | | | | Cu (mg/L) | | | | |
| --- | --- | --- | --- | --- | --- | --- | --- | --- | --- | --- |
|  | 100 | 200 | 300 | 400 | 500 | 100 | 200 | 300 | 400 | 500 |
| **S1** | + | + | + | + | - | + | + | + | + | + |
| **S2** | + | + | + | - | - | + | + | + | + | - |
| S3 | + | + | - | - | - | + | + | + | + | - |
| **S4** | + | + | + | - | - | + | + | + | + | + |
| **S5** | + | + | + | + | + | + | + | + | + | + |
| **S6** | + | + | + | + | - | + | + | + | + | + |
| **S7** | + | + | + | - | - | + | + | + | + | - |
| S8 | + | + | - | - | - | + | + | + | - | - |
| **S9** | + | + | + | + | - | + | + | + | + | + |
| S10 | + | + | - | - | - | + | + | + | + | - |
| **S11** | + | + | + | - | - | + | + | + | + | + |
| S12 | + | + | - | - | - | + | + | + | + | - |
| **S13** | + | + | + | - | - | + | + | + | + | + |
| **S14** | + | + | + | - | - | + | + | + | + | + |
| **S15** | + | + | + | - | - | + | + | + | + | - |
| **S16** | + | + | + | + | - | + | + | + | + | + |
| **S17** | + | + | + | + | + | + | + | + | + | + |
| **S18** | + | + | + | - | - | + | + | + | + | + |
| S19 | + | + | - | - | - | + | + | + | - | - |
| S20 | + | + | - | - | - | + | + | + | + | - |
| **S21** | + | + | + | + | - | + | + | + | + | + |
| S22 | + | + | - | - | - | + | + | + | + | - |
| **S23** | + | + | + | + | + | + | + | + | + | + |
| S24 | + | + | - | - | - | + | + | + | + | - |
| **S25** | + | + | + | + | + | + | + | + | + | + |
| **S26** | + | + | + | + | + | + | + | + | + | + |
| **S27** | + | + | + | + | - | + | + | + | + | + |
| **S28** | + | + | + | + | - | + | + | + | + | + |
| **S29** | + | + | + | + | + | + | + | + | + | + |
| **S30** | + | + | + | + | + | + | + | + | + | + |
| S31 | + | + | - | - | - | + | + | + | - | - |
| **S32** | + | + | + | - | - | + | + | + | + | - |
| **S33** | + | + | + | - | - | + | + | + | + | + |
| **S34** | + | + | + | + | - | + | + | + | + | - |
| **S35** | + | + | + | + | + | + | + | + | + | - |
| S36 | + | + | - | - | - | + | + | + | + | - |
| **S37** | + | + | + | + | + | + | + | + | + | + |
| **S38** | + | + | + | + | + | + | + | + | + | + |
| **S39** | + | + | + | + | - | + | + | + | + | - |
| **S40** | + | + | + | + | + | + | + | + | + | + |
| **S41** | + | + | + | + | - | + | + | + | + | - |
| **S42** | + | + | + | + | + | + | + | + | + | + |
| **S43** | + | + | + | + | + | + | + | + | + | + |
| **S44** | + | + | + | + | + | + | + | + | + | + |
| **S45** | + | + | + | + | + | + | + | + | + | + |
| **S46** | + | + | + | + | - | + | + | + | + | + |
| **S47** | + | + | + | + | + | + | + | + | + | + |
| S48 | + | + | - | - | - | + | + | + | - | - |
| **S49** | + | + | + | - | - | + | + | + | + | + |
| **S50** | + | + | + | + | + | + | + | + | + | + |
| S51 | + | + | - | - | - | + | + | + | - | - |
| **S52** | + | + | + | + | - | + | + | + | + | + |
| **S53** | + | + | + | + | + | + | + | + | + | + |
| **S54** | + | + | + | + | + | + | + | + | + | + |
| S55 | + | + | - | - | - | + | + | + | + | - |
| S56 | + | + | - | - | - | + | + | + | - | - |
| **S57** | + | + | + | + | + | + | + | + | + | + |
| **S58** | + | + | + | + | + | + | + | + | + | + |
| **S59** | + | + | + | + | + | + | + | + | + | + |
| **S60** | + | + | + | - | - | + | + | + | - | - |

Notes:

+, positive growth; -, no growth.

The isolates marked in bold were able to resist 300 mg·L^-1^ Zn and 300 mg·L^-1^ Cu simultaneously.
