# Supplementary material for: Screening and Evaluation of the Bioremediation Potential of Cu/Zn-Resistant, Autochthonous Acinetobacter sp. FQ-44 from Sonchus oleraceus L
Source: Front Plant Sci. 2016 Sep 30;7:1487. doi: 10.3389/fpls.2016.01487 (PMC5043060; doi:10.3389/fpls.2016.01487)
Supplement: Supplementary file 2 [file Table_2.DOCX]

**Table S2. PGP characteristics of all preliminarily selected Cu/Zn-resistant strains.**

| Isolates | IAA synthesis  (mg·L^-1^) | Siderophore  production  (A/A_r_)^a^ | Phosphate  solubilization  (mg·L^-1^)^b^ | ACC deaminase  (nmol α-KB  mg^-1^·protein·h^-1^) |
| --- | --- | --- | --- | --- |
| S2 | 1.09 ± 0.14 | 0.48 ± 0.08 | 32.88 ± 1.55 | 104.23 ± 6.31 |
| S4 | 1.35 ± 0.12 | 0.41 ± 0.09 | 21.19 ± 0.49 | 85.25 ± 4.17 |
| S7 | 1.46 ± 0.11 | 0.48 ± 0.13 | 24.81 ± 1.61 | 87.42 ± 2.46 |
| S11 | 1.63 ± 0.06 | 0.78 ± 0.13 | 24.12 ± 1.51 | 91.47 ± 2.25 |
| S13 | 1.43 ± 0.09 | 0.51 ± 0.01 | 32.51 ± 1.45 | 85.25 ± 4.17 |
| S18 | 1.41 ± 0.18 | 0.73 ± 0.09 | 32.79 ± 1.07 | 80.42 ± 3.14 |
| S21 | 10.55 ± 0.08 | 0.13 ± 0.01 | 53.34 ± 1.29 | 83.55 ± 5.75 |
| S23 | 6.48 ± 0.24 | 0.29 ± 0.01 | 34.21 ± 1.06 | 86.25 ± 5.67 |
| S25 | 7.01 ± 0.58 | 0.40 ± 0.09 | 36.36 ± 1.41 | 69.50 ± 1.99 |
| S26 | 3.45 ± 0.29 | 0.25 ± 0.05 | 35.00 ± 0.96 | 83.32 ± 5.45 |
| S29 | 4.42 ± 0.15 | 0.10 ± 0.01 | 39.42 ± 1.38 | 74.77 ± 7.25 |
| S30 | 5.10 ± 0.16 | 0.12 ± 0.05 | 35.45 ± 1.06 | 110.36 ± 6.26 |
| S34 | 1.13 ± 0.11 | 0.61 ± 0.01 | 13.43 ± 0.51 | 71.25 ± 4.43 |
| S42 | 20.17 ± 0.26 | 0.34 ± 0.09 | 39.76 ± 0.82 | 74.23 ± 0.44 |
| S44 | 29.57 ± 0.95 | 0.29 ± 0.04 | 74.75 ± 1.48 | 80.24 ± 3.85 |
| S45 | 25.15 ± 0.56 | 0.34 ± 0.02 | 55.38 ± 1.41 | 78.41 ± 5.44 |
| S53 | 3.12 ± 0.22 | 0.50 ± 0.04 | 28.38 ± 0.51 | 66.73 ± 3.39 |
| S54 | 2.91 ± 0.14 | 0.74 ± 0.02 | 25.49 ± 0.55 | 68.54 ± 2.83 |
| S57 | 9.81 ± 0.20 | 0.23 ± 0.02 | 55.81 ± 1.55 | 65.22 ± 4.33 |

Notes:

Average ± standard error from three samples.

^a^ Siderophore production: little, 0.8-1.0; low, 0.6-0.8; moderate, 0.4-0.6; high, 0.2-0.4; very high, 0-0.2.

^b^ Concentration of phosphorus.
