# Supplementary material for: Screening and Evaluation of the Bioremediation Potential of Cu/Zn-Resistant, Autochthonous Acinetobacter sp. FQ-44 from Sonchus oleraceus L
Source: Front Plant Sci. 2016 Sep 30;7:1487. doi: 10.3389/fpls.2016.01487 (PMC5043060; doi:10.3389/fpls.2016.01487)
Supplement: Supplementary file 3 [file Table_3.DOCX]

| Correlation | | plant growth parameters | | |
| --- | --- | --- | --- | --- |
|  |  | Root length | Shoot length | Fresh weight |
| PGP traits | IAA | r = 0.41,  *p* = 0.24 | r = 0.23,  *p* = 0.52 | r = 0.70,  *p* = 0.02 |
|  | Siderphores | r = 0.07,  *p* = 0.84 | r = 0.02,  *p* = 0.97 | r =0.06,  *p* = 0.86 |
|  | Phosphate solubilization | r = 0.45,  *p* = 0.19 | r = 0.18,  *p* =0.63 | r = 0.51,  *p* = 0.13 |

**Table S3.** **Correlations between PGP traits and plant growth parameters.**
